# Factor Analysis: a means for theory and instrument development in support of construct validity

**DOI:** 10.5116/ijme.5f96.0f4a

**Published:** 2020-11-06

**Authors:** Mohsen Tavakol, Angela Wetzel

**Affiliations:** 1 School of Medicine, Medical Education Centre, the University of Nottingham, UK; 2 School of Education, Virginia Commonwealth University, USA

**Keywords:** Factor Analysis, theory and instrument development, construct validity

## Introduction

Factor analysis (FA) allows us to simplify a set of complex variables or items using statistical procedures to explore the underlying dimensions that explain the relationships between the multiple variables/items. For example, to explore inter-item relationships for a 20-item instrument, a basic analysis would produce 400 correlations; it is not an easy task to keep these matrices in our heads. FA simplifies a matrix of correlations so a researcher can more easily understand the relationship between items in a scale and the underlying factors that the items may have in common. FA is a commonly applied and widely promoted procedure for developing and refining clinical assessment instruments to produce evidence for the construct validity of the measure.

In the literature, the strong association between construct validity and FA is well documented, as the method provides evidence based on test content and evidence based on internal structure, key components of construct validity.^1^ From FA, evidence based on internal structure and evidence based on test content can be examined to tell us what the instrument really measures - the intended abstract concept (i.e., a factor/dimension/construct) or something else. Establishing construct validity for the interpretations from a measure is critical to high quality assessment and subsequent research using outcomes data from the measure. Therefore, FA should be a researcher’s best friend during the development and validation of a new measure or when adapting a measure to a new population. FA is also a useful companion when critiquing existing measures for application in research or assessment practice. However, despite the popularity of FA, when applied in medical education instrument development, factor analytic procedures do not always match best practice.^2^ This editorial article is designed to help medical educators use FA appropriately.

### The Applications of FA

The applications of FA depend on the purpose of the research. Generally speaking, there are two most important types of FA: Explorator Factor Analysis (EFA) and Confirmatory Factor Analysis (CFA).

### Exploratory Factor Analysis

Exploratory Factor Analysis (EFA) is widely used in medical education research in the early phases of instrument development, specifically for measures of latent variables that cannot be assessed directly. Typically, in EFA, the researcher, through a review of the literature and engagement with content experts, selects as many instrument items as necessary to fully represent the latent construct (e.g., professionalism). Then, using EFA, the researcher explores the results of factor loadings, along with other criteria (e.g., previous theory, Minimum average partial,^3^ Parallel analysis,^4^ conceptual meaningfulness, etc.) to refine the measure. Suppose an instrument consisting of 30 questions yields two factors - Factor 1 and Factor 2. A good definition of a factor as a theoretical construct is to look at its factor loadings.^5^ The factor loading is the correlation between the item and the factor; a factor loading of more than 0.30 usually indicates a moderate correlation between the item and the factor. Most statistical software, such as SAS, SPSS and R, provide factor loadings. Upon review of the items loading on each factor, the researcher identifies two distinct constructs, with items loading on Factor 1 all related to professionalism, and items loading on Factor 2 related, instead, to leadership. Here, EFA helps the researcher build evidence based on internal structure by retaining only those items with appropriately high loadings on Factor 1 for professionalism, the construct of interest.

It is important to note that, often, Principal Component Analysis (PCA) is applied and described, in error, as exploratory factor analysis.^2^^,^^6^ PCA is appropriate if the study primarily aims to reduce the number of original items in the intended instrument to a smaller set.^7^ However, if the instrument is being designed to measure a latent construct, EFA, using Maximum Likelihood (ML) or Principal Axis Factoring (PAF), is the appropriate method.^7^  These exploratory procedures statistically analyze the interrelationships between the instrument items and domains to uncover the unknown underlying factorial structure (dimensions) of the construct of interest. PCA, by design, seeks to explain total variance (i.e., specific and error variance) in the correlation matrix. The sum of the squared loadings on a factor matrix for a particular item indicates the proportion of variance for that given item that is explained by the factors. This is called the communality. The higher the communality value, the more the extracted factors explain the variance of the item. Further, the mean score for the sum of the squared factor loadings specifies the proportion of variance explained by each factor. For example, assume four items of an instrument have produced Factor 1, factor loadings of Factor 1 are 0.86, 0.75, 0.66 and 0.58, respectively. If you square the factor loading of items, you will get the percentage of the variance of that item which is explained by Factor 1. In this example, the first principal component (PC) for item1, item2, item3 and item4 is 74%, 56%, 43% and 33%, respectively. If you sum the squared factor loadings of Factor 1, you will get the eigenvalue, which is 2.1 and dividing the eigenvalue by four (2.1/4= 0.52) we will get the proportion of variance accounted for Factor 1, which is 52 %. Since PCA does not separate specific variance and error variance, it often inflates factor loadings and limits the potential for the factor structure to be generalized and applied with other samples in subsequent study. On the other hand, Maximum likelihood and Principal Axis Factoring extraction methods separate common and unique variance (specific and error variance), which overcomes the issue attached to PCA.  Thus, the proportion of variance explained by an extracted factor more precisely reflects the extent to which the latent construct is measured by the instrument items. This focus on shared variance among items explained by the underlying factor, particularly during instrument development, helps the researcher understand the extent to which a measure captures the intended construct. It is useful to mention that in PAF, the initial communalities are not set at 1s, but they are chosen based on the squared multiple correlation coefficient. Indeed, if you run a multiple regression to predict say  item1 (dependent variable)  from other items (independent variables) and then look at the R-squared (R2), you will see R2 is equal to the communalities of item1 derived from PAF.

### Confirmatory Factor Analysis

When prior EFA studies are available for your intended instrument, Confirmatory Factor Analysis extends on those findings, allowing you to confirm or disconfirm the underlying factor structures, or dimensions, extracted in prior research. CFA is a theory or model-driven approach that tests how well the data “fit” to the proposed model or theory. CFA thus departs from EFA in that researchers must first identify a factor model before analysing the data. More fundamentally, CFA is a means for statistically testing the internal structure of instruments and relies on the maximum likelihood estimation (MLE) and a different set of standards for assessing the suitability of the construct of interest.^7^^,^^8^

 Factor analysts usually use the path diagram to show the theoretical and hypothesized relationships between items and the factors to create a hypothetical model to test using the ML method. In the path diagram, circles or ovals represent factors. A rectangle represents the instrument items. Lines (→ or ↔) represent relationships between items. No line, no relationship. A single-headed arrow shows the causal relationship (the variable that the arrowhead refers to is the dependent variable), and a double-headed shows a covariance between variables or factors.

If CFA indicates the primary factors, or first-order factors, produced by the prior PAF are correlated, then the second-order factors need to be modelled and estimated to get a greater understanding of the data. It should be noted if the prior EFA applied an orthogonal rotation to the factor solution, the factors produced would be uncorrelated. Hence, the analysis of the second-order factors is not possible. Generally, in social science research, most constructs assume inter-related factors, and therefore should apply an oblique rotation. The justification for analyzing the second-order factors is that when the correlations between the primary factors exist, CFA can then statistically model a broad picture of factors not captured by the primary factors (i.e., the first-order factors).^9^  The analysis of the first-order factors is like surveying mountains with a zoom lens binoculars, while the analysis of the second-order factors uses a wide-angle lens.^10^ Goodness of- fit- tests need to be conducted when evaluating the hypothetical model tested by CFA. The question is: does the new data fit the hypothetical model? However, the statistical models of the goodness of- fit- tests are complex, and extend beyond the scope of this editorial paper; thus,we strongly encourage the readers consult with factors analysts to receive resources and possible advise.

## Conclusions

Factor analysis methods can be incredibly useful tools for researchers attempting to establish high quality measures of those constructs not directly observed and captured by observation. Specifically, the factor solution derived from an Exploratory Factor Analysis provides a snapshot of the statistical relationships of the key behaviors, attitudes, and dispositions of the construct of interest. This snapshot provides critical evidence for the validity of the measure based on the fit of the test content to the theoretical framework that underlies the construct. Further, the relationships between factors, which can be explored with EFA and confirmed with CFA, help researchers interpret the theoretical connections between underlying dimensions of a construct and even extending to relationships across constructs in a broader theoretical model. However, studies that do not apply recommended extraction, rotation, and interpretation in FA risk drawing faulty conclusions about the validity of a measure. As measures are picked up by other researchers and applied in experimental designs, or by practitioners as assessments in practice, application of measures with subpar evidence for validity produces a ripple effect across the field. It is incumbent on researchers to ensure best practices are applied or engage with methodologists to support and consult where there are gaps in knowledge of methods. Further, it remains important to also critically evaluate measures selected for research and practice, focusing on those that demonstrate alignment with best practice for FA and instrument development.^7^^, ^^11^

### Conflicts of Interest

The authors declare that they have no conflicts of interest.
